# Trends in Decarceration, COVID-19 Cases, and SARS-CoV-2 Testing in US Immigration Detention Centers From September 2020 to August 2021

**DOI:** 10.1001/jamanetworkopen.2021.48859

**Published:** 2022-02-16

**Authors:** Nishant Uppal, Elizabeth T. Chin, Parsa Erfani, Raquel Sofia Sandoval, Caroline H. Lee, Ranit Mishori, Katherine R. Peeler

**Affiliations:** 1 Harvard Medical School, Boston, Massachusetts; 2 Department of Biomedical Data Science, Stanford University, Palo Alto, California; 3 Georgetown University School of Medicine, Washington, DC; 4 Division of Medical Critical Care, Boston Children’s Hospital, Boston, Massachusetts

## Abstract

This cohort study compares COVID-19 case and testing rates in US Immigration and Customs Enforcement detention centers with rates in the US population from September 2020 through August 2021.

## Introduction

Throughout the COVID-19 pandemic, the number of migrants detained by US Immigration and Customs Enforcement (ICE) has fluctuated widely.^1^ During certain periods, ICE reduced its population to mitigate the risk of SARS-CoV-2 transmission among detained migrants, but federal oversight revealed inconsistent use of preventative measures across facilities.^2^ We assessed trends in COVID-19 cases and testing in ICE detention centers as they relate to changes in the detained population size.

## Methods

We performed a retrospective, population-based cohort study of reported COVID-19 cases and tests conducted in ICE detention centers. The study followed the Strengthening the Reporting of Observational Studies in Epidemiology (STROBE) reporting guideline. Data from September 2020 through August 2021 were obtained from the Vera Institute, which aggregates reporting from ICE’s COVID-19 website.^3^ The Harvard Medical School institutional review board waived review because this cohort study used public, deidentified data and was determined not to constitute human participants research.

Monthly case and testing rates in ICE detention centers were calculated for the study period using the cumulative totals of daily COVID-19 cases, SARS-CoV-2 tests, and monthly detained population across centers.^1^ Monthly case and testing rates in the US were calculated for the study period using data from the US Centers for Disease Control and Prevention and the US Census Bureau for states containing ICE detention facilities. Monthly rate ratios for ICE compared with the US population were calculated. Point estimates and ranges were calculated using monthly statistics weighted by the number of days per month. Statistics for cases and tests were standardized to 30-day counts and rates. Details are provided in the eMethods in the Supplement. Data were analyzed using R, version 4.0.2 (R Project for Statistical Computing).

## Results

The mean monthly population detained by ICE decreased by 30.8% from September 2020 (20 365 individuals) to February 2021 (14 084 individuals) and subsequently increased by 79.6% in August 2021 (25 299 individuals).^1^ Trends in the mean monthly detained population, monthly case rates, monthly test rates, and rate ratios (ICE-to-US general population) are shown in the Figure.

**Figure.**
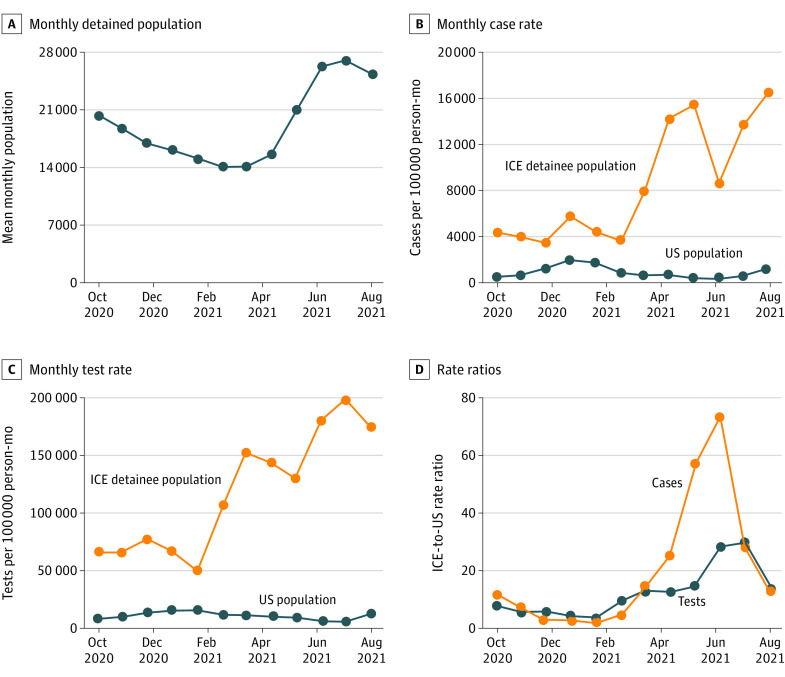
Trends in COVID-19 Case Rates, Testing Rates, and Rate Ratios in US Immigration and Customs Enforcement (ICE) Detention Centers and the State-Matched US Population From September 2020 to August 2021

During the 3-month period when the detained population was at its highest (June to August 2021), the mean ICE monthly case rate was 12 660 per 100 000 person-months (range, 8551-16 020 per 100 000 person-months) and the test rate was 180 783 per 100 000 person-months (range, 168 447-193 734 per 100 000 person-months) (Table). Concurrently, the mean US monthly case rate was 608 per 100 000 person-months (range, 117-1215 per 100 000 person-months) and test rate was 8499 per 100 000 person-months (range, 6389-12 445 per 100 000 person-months).

**Table.** Monthly COVID-19 Case Rates, Test Rates, and Rate Ratios From September 2020 to August 2021

	Mean (range)			
	September 2020 to November 2020	December 2020 to February 2021	March 2021 to May 2021	June 2021 to August 2021
ICE detention				
Monthly population	18 661 (16 870-20 365)	15 137 (14 610-15 608)	16 903 (13 692-20 284)	26 184 (24 483-26 196)
Reported COVID-19 cases, No. per mo	716 (589-853)	702 (552-898)	2141 (1075-3131)	3306 (2240-4053)
Reported individuals tested for COVID-19, No. per mo	12 775 (11 846-13 478)	11 058 (7439-16 062)	23 225 (20 882-26 304)	47 412 (42 615-52 419)
Monthly case rate, per 100 000 person-mo	3816 (3491-4189)	4589 (3918-5569)	12 232 (7599-14 936)	12 660 (8551-16 020)
Monthly test rate, per 100 000 person-mo	68 809 (63 200-77 374)	73 882 (49 275-114 043)	138 997 (125 497-147 596)	180 783 (168 447-193 734)
Test positivity rate, %	5.6 (4.5-6.3)	6.9 (3.7-8.3)	9.0 (5.0-11.5)	7.0 (4.8-9.2)
State-matched US population				
Monthly case rate, per 100 000 person-mo	751 (360-1365)	1527 (762-1868)	450 (260-560)	608 (117-1215)
Monthly test rate, per 100 000 person-mo	10 983 (8353-14 219)	14 614 (11 845-16 198)	10 528 (8595-11 572)	8499 (6389-12 445)
Test positivity rate, %	6.4 (4.3-9.6)	10.1 (6.9-11.5)	4.2 (2.9-4.8)	6.3 (1.8-9.4)
Monthly ICE-to-US case rate ratio	7.1 (2.6-11.6)	3.4 (2.2-5.5)	32.4 (13.7-55.6)	37.9 (12.8-73.3)
Monthly ICE-to-US test rate ratio	6.5 (5.4-7.9)	5.5 (3.1-10.3)	13.3 (12.4-14.1)	23.8 (13.1-28.7)

Abbreviation: ICE, US Immigration and Customs Enforcement.

During the months of recarceration, the mean ICE-to-US monthly case rate ratio was 32.4 (range, 13.7-55.6) from March through May 2021 and 37.9 (range, 12.8-73.3) from June through August 2021 (Table). The mean test positivity rate was 9.0% (range, 5.0%-11.5%) in ICE detention and 4.2% (range, 2.9%-4.8%) in the US from March through May 2021; the mean test positivity rate was 7.0% (range, 4.8%-9.2%) in ICE detention and 6.3% (range, 1.8%-9.4%) in the US from June through August 2021. The mean ICE-to-US monthly test rate ratio was 13.3 (range, 12.4-14.1) from March through May 2021 and 23.8 (range, 13.1-28.7) from June through August 2021.

## Discussion

Prior analyses of COVID-19 cases in ICE detention centers found that mean monthly case rates from April through August 2020 increased 4.4-fold despite a decreasing detained population.^1,4^ In the present study, as decarceration efforts continued from September 2020 through February 2021, ICE case rates were a mean 5.3-fold higher and test rates were a mean 6.5-fold higher than rates for the state-matched US population. As the detained population size increased from January to August 2021, the mean monthly case rate ratio increased by a factor of 6.7 and test rate ratio increased by a factor of 3.1 compared the prior 6 months.

An increasing detained population from March through July 2021 may have coincided with higher rates of screening tests for asymptomatic individuals in ICE detention centers. Nevertheless, test positivity rates were higher compared with the general US population, where testing protocols were variable.

Limitations of the study include relying on ICE’s publicly available data, which may be subject to reporting delays and missing components. Comparison of rates between detained migrants and the state-matched US population was limited by differences in testing and reporting methods. Estimates of COVID-19 cases in ICE detention centers were limited by the lack of available susceptibility data (ie, rates of prior infection and vaccination rates) and may have been overestimated owing to higher rates of routine testing of asymptomatic individuals in ICE detention centers compared with rates in the US population. Testing rates were limited based on the number of detained individuals being retested for SARS-CoV-2 because individual-level data and variance in the sensitivity and specificity of COVID-19 assays were not available.

This cohort study highlights the importance of renewed COVID-19 mitigation efforts in detention facilities, which experience continued challenges in adhering to social distancing restrictions, achieving high vaccination rates, and instituting mass screening and isolation protocols.^2^ A reduction in ICE’s detained population may be associated with limiting further spread of COVID-19 in detention centers because decarceration has been associated with reduced COVID-19 incidence in other carceral settings.^5,6^

## Notes

Supplement.
eMethods.
