# Supplementary material for: Trends in Decarceration, COVID-19 Cases, and SARS-CoV-2 Testing in US Immigration Detention Centers From September 2020 to August 2021
Source: JAMA Netw Open. 2022 Feb 16;5(2):e2148859. doi: 10.1001/jamanetworkopen.2021.48859 (PMC8851301; doi:10.1001/jamanetworkopen.2021.48859)
Supplement: Supplement. — eMethods. [file jamanetwopen-e2148859-s001.pdf]

Supplemental Online Content

Uppal N, Chin ET, Erfani P, et al. Trends in decarceration, COVID-19 cases, and SARS-CoV-2 testing in US immigration detention centers from September 2020 to August 2021. *JAMA Netw Open.* 2022;5(2):e2148859.
doi:10.1001/jamanetworkopen.2021.48859

eMethods.

This supplemental material has been provided by the authors to give readers additional information about their work.

© 2022 Uppal N et al. *JAMA Network Open.*

eMethods

Calculation of Monthly Case Rates and Monthly Testing Rates in ICE Detention

COVID-19 case data, SARS-CoV-2 testing data, and the detained population size in US Immigration and Customs Enforcement (ICE) detention facilities was extracted from the Vera Institute.[1] Data on the average detained population size each month was extracted from ICE.[2] Confirmed cases were provided as a daily cumulative total of detainees who have tested positive for COVID-19 while in ICE custody since testing began in February 2020. Tests were provided as a daily cumulative total of the number of detainees who were tested for SARS-CoV-2. The numbers of monthly cases and monthly tests were calculated as the differences between cumulative totals at the end of each month. A validity check was performed by using internet archives to extract historical data on cumulative monthly case and test totals from ICE.[3] This revealed an average monthly margin of error of 0 percent between both case and testing data from the Vera Institute and data from ICE.

Monthly case rates were calculated as the total number of COVID-19 cases each month divided by the average population size and then multiplied by 100,000 to report on a per 100,000 person-months basis. Monthly testing rates were calculated as the total number of COVID-19 tests each month divided by the average population size and then multiplied by 100,000 to report on a per 100,000 person-months basis. Point estimates and min-max intervals were calculated using monthly statistics weighted by the number of days per month. Statistics for cases and tests were standardized to 30-day counts and rates.

Calculation of Monthly Case Rates and Monthly Testing Rates in the United States

The states containing ICE detention facilities were identified using ICE's publicly available listing of detention facilities.[4] These 29 states included Alabama, Arizona, California, Colorado, Florida, Georgia, Illinois, Indiana, Iowa, Kentucky, Louisiana, Maryland, Massachusetts, Michigan, Minnesota, Mississippi, Missouri, Nebraska, New Jersey, New Mexico, New York, Ohio, Oklahoma, Pennsylvania, Texas, Utah, Virginia, Washington, and Wisconsin. COVID-19 case data and SARS-CoV-2 testing data for these states from September 2020 to August 2021 were extracted from the Centers for Disease Control and Prevention[5,6] and then aggregated on a monthly basis. The most recent data on state population sizes for 2019 was extracted for these states from the Census Bureau[7] and then aggregated. Monthly case rates were calculated as the total number of COVID-19 cases across all included states divided by the aggregated population size and then multiplied by 100,000 to report on a per 100,000 person-months basis. Monthly testing rates were calculated as the total number of COVID-19 tests across all included states divided by the aggregated population size and then multiplied by 100,000 to report on a per 100,000 person-months basis. Point estimates and min-max intervals were calculated using monthly statistics weighted by the number of days per month. Statistics for cases and tests were standardized to 30-day counts and rates.

© 2022 Uppal N et al. *JAMA Network Open.*

eReferences

1. Vera Institute. Tracking COVID-19 in immigration detention. Accessed October 14, 2021. https://www.vera.org/tracking-covid-19-in-immigration-detention
2. US Immigration and Customs Enforcement. Detention management. Accessed October 14, 2021. https://www.ice.gov/detain/detention-management
3. US Immigration and Customs Enforcement. ICE Guidance on COVID-19. Accessed December 22, 2021. https://www.ice.gov/coronavirus
4. US Immigration and Customs Enforcement. Detention Facilities. Accessed December 21, 2021. https://www.ice.gov/detention-facilities
5. Centers for Disease Control and Prevention. United States COVID-19 Cases and Deaths by State over Time. Accessed December 21, 2021. https://data.cdc.gov/Case-Surveillance/United-States-COVID-19-Cases-and-Deaths-by-State-o/9mfq-cb36
6. Centers for Disease Control and Prevention. COVID Data Tracker, Trends in Number of COVID-19 Cases and Deaths in the US Reported to CDC, by State/Territory. Accessed December 21, 2021. https://covid.cdc.gov/covid-data-tracker/#trends_newtestresultsreported
7. United States Census Bureau. State Population Totals and Components of Change: 2010-2019. Accessed December 21, 2021. https://www.census.gov/data/tables/time-series/demo/popest/2010s-state-total.html

© 2022 Uppal N et al. *JAMA Network Open.*
